# 9th Nci-Eortc Symposium on New Drugs in Cancer Therapy

**DOI:** 10.1155/MBD.1995.125

**Published:** 1995


					9th NCI-EORTC SYMPOSIUM ON NEW DRUGS
IN CANCER THERAPY
March 12-15,1996
Amsterdam, The Netherlands
The series of NCI-EORTC Symposia on New drugs in Cancer Therapy has been receiving increasing
appreciation and recognition. This bi-annual "Amsterdam New Drug Meeting" is now perceived by many as one
of the most exciting conferences in the field of new anticancer drug development. It is therefore a pleasure to
announce that preparations for the next NCI-EORTC Symposium have been initiated. The Organizing
Committee invites you to reserve March 12-15, 1996 for the 9th NCI EORTC Symposium on New Drugs in
Cancer Therapy.
International organizing committee
Symposium president: H.M. Pinedo
J. P. Armand
B. A. Chabner
T. A. Connors
G. A. Curt
M. D'lncalci
M. A. Friedman
H. H. Hansen
R. E. C. Henrar
C. K. van Kalken
M. W. Lobbezoo
D. L. Longo
J. G. McVie
D. Shoemaker
D. Parkinson
J. B. Vermorken
J. Verweij
O. C. Yoder
Amsterdam, The Netherlands
Villejuif, France
Bethesda, U.S.A.
Carshalton, U.K.
Bethesda, U.S.A.
Milano, Italy
Bethesda, U.S.A.
Copenhagen, Denmark
Amsterdam, The Netherlands
Amsterdam, The Netherlands
Amsterdam, The Netherlands
Bethesda, U.S.A.
London, United Kingdom
Bethesda, U.S.A.
Bethesda, U.S.A.
Amsterdam, The Netherlands
Rotterdam, The Netherlands
Brussels, Belgium
Scientific advisory board
C. J. Allegra
S. G. Arbuck
J. H. Beijnen
J. R. Bertino
B. Chevallier
A. H. Calvert
M. Christian
R. C. Donehower
Ch. Dittrich
J. Double
G. Eisenbrand
E. A. Eisenhauer
H. H. Fiebig
G. Giaccone
C. K. Grieshaber
K. K. Harrap
H. R. Hendriks
J. Mendelsohn
C. E. Myers
M. Ogawa
I. Pastan
M. Potmesil
E. A. Sausville
S. Seeber
F. Valeriote
D. D. Von Hoff
J. Wagstaff
R. E. Wittes
H. Zwierzina
Bethesda, U.S.A.
Bethesda. U.S.A.
Amsterdam, The Netherlands
New York, U.S.A.
Rouen, France
Newcastle, U.K.
Bethesda, U.S.A.
Baltimore, U.S.A.
Vienna, Austria
Bradford, U.K.
Kaiserslautern, Germany
Kingston, Canada
Freiburg, Germany
Amsterdam, The Netherlands
Rockville, U.S.A.
Sutton, U.K.
Amsterdam, The Netherlands
New York, U.S.A.
Richmond, U.S.A.
Tokyo, Japan
Bethesda, U.S.A.
New York, U.S.A.
Bethesda, U.S.A.
Essen, Germany
Detroit, U.S.A.
San Antonio, U.S.A.
Amsterdam, The Netherlands
Bethesda, U.S.A.
Innsbruck, Austria
125

				

